# Enhancing Life’s Essential 8 for Cardiovascular Risk Assessment in Breast Cancer Survivors

**DOI:** 10.1016/j.jaccao.2024.09.010

**Published:** 2024-11-19

**Authors:** Shanshan Gao, Jingjjing Mu, Beina Hui, Weibin Hu, Yongkai Lu

We read with great interest the paper by Wadden et al^1^ on the association between the Life’s Essential 8 (LE8) score and incident cardiovascular disease (CVD) in U.S. women with breast cancer. The study highlights the crucial role of cardiovascular health in this population, demonstrating that higher LE8 scores were associated with reduced CVD risk. However, there are additional considerations that could further enhance the study’s impact.

Although the LE8 score effectively captures modifiable lifestyle factors, the study did not sufficiently consider cancer-specific factors such as the type, dose, and duration of cardiotoxic therapies.^2^ Given the cardiotoxicity of treatments such as anthracyclines and trastuzumab, incorporating these into the LE8 model could provide a more accurate risk stratification tool. This could then enable targeted cardiovascular interventions for elevated risk breast cancer survivors.

Furthermore, the study’s reliance on self-reported data for several LE8 components (eg, diet, physical activity) may introduce biases.^3^ Future research should consider using objective measures, such as wearable devices, to monitor physical activity and sleep patterns continuously. This approach would enhance data reliability and allow dynamic, personalized interventions.

Additionally, integrating biomarkers beyond the current LE8 metrics could improve risk assessment. Inflammatory markers such as C-reactive protein and interleukin-6 have been linked to cardiovascular risk in patients with breast cancer.^4^ Including these in the LE8 framework could help detect subclinical CVD risks and facilitate early intervention.

Last, although the study focuses on a U.S. cohort, validating the LE8 tool in diverse populations is essential. Cardiovascular risk factors and treatment protocols vary significantly worldwide.^5^ Conducting similar studies across different environments could determine the external validity of the LE8 score and necessary regional adaptations.

In conclusion, Wadden et al^1^ provide a valuable foundation for understanding cardiovascular health in breast cancer survivors. Expanding the model to include treatment factors, objective data collection, additional biomarkers, and global validation could enhance its utility, ultimately improving cardiovascular outcomes for breast cancer survivors.
